# Acknowledgement to Reviewers of *Marine Drugs* in 2018

**DOI:** 10.3390/md17010059

**Published:** 2019-01-16

**Authors:** Marine Drugs Editorial Office

**Affiliations:** MDPI, St. Alban-Anlage 66, 4052 Basel, Switzerland

Rigorous peer-review is the corner-stone of high-quality academic publishing. The editorial team greatly appreciates the reviewers who contributed their knowledge and expertise to the journal’s editorial process over the past 12 months. In 2018, a total of 535 papers were published in the journal, with a median time to first decision of 16.52 days and a median time to publication of 33 days. The editors would like to express their sincere gratitude to the following reviewers for their cooperation and dedication in 2018:

Abdel-Sayed, PhilippeLiang, ZhibinAberturas, JavierLiaw, Chih-ChuangAbeywardana, TharindumalaLiguori, AngeloAbou Neel, Ensanya A.Lin, Hai-ShuAccoroni, StefanoLindstrom, Jon MartinAdunyah, Samuel E.Lionetti, LillàAfonso, Clélia N.Liu, Shing-HwaAgarwal, VinayakLiu, XiaoxiAgyei, DominicLiu, YaminAires, AlfredoLiu, YongqiangAlexopoulos, AthanassiosLivaniou, EvangeliaAl-Fatimi, MohamedLiverani, LilianaAlonso, EvaLo Giudice, AngelinaÁlvarez, MercedesLo, Kai-YinAmatriain, DanielLoret, Erwann P.Amillis, SotirisLowe, EdwardAminin, Dmitry L.Lu, JunAmsberg, Gunhild VonLucia Tornesello, AnnaAnadón, ArturoLudwiczuk, AgnieszkaAnas, Andrea RoxanneLundstrom, KennethAnastyuk, Stanislav D.Lupiañez, José AntonioAndolfi, AnnaLuvisetto, SiroAndrade, Paula BMa, YongAndreev, KonstantinMa, ZexuAndrianasolo, EricMacone, AlbertoAnraku, MakotoMacrander, JasonApone, FabioMaddau, LuciaAppendino, GiovanniMaeda, HayatoArakawa, OsamuMaehre, HanneAranaz, InmaculadaMagarlamov, Timur Yu.Ariga, KatsuhikoMaher, PamelaArmanini, DecioMakishima, MakotoArnason, JohnMalik, RavinderAsai, DaisukeMallipeddi, Prema LathaAshton, TrentMancini, InesAvarvari, NarcisManconi, MariaAvila, ConxitaMandrich, LuigiAviles, Francesc XavierMandrone, ManuelaAykin-burns, NukhetManfrin, C.Azuma, KazuoMangoni, Maria LuisaBabo, PedroManjunath, ManuboluBabu, AnishMann, FrancisBaerga-Ortiz, AbelMaoka, TakashiBahn, AndrewMarcal, HelderBai, RenrenMarengo, BarbaraBaker, BillMargassery, Lekha MenonBaki, GabriellaMarino, AngelaBakunina, IrinaMariottini, Gian LuigiBalasubramaniam, MeenakshisundaramMarrero, Ana R. DíazBallottari, MatteoMartin, JessicaBaniahmad, AriaMartini, ClaudiaBanskota, Arjun H.Martini, DanielaBarba, Francisco J.Martins, AlbinoBarbaglio, AliceMartins, NatáliaBarelle, CarolineMarto, JoanaBarnathan, GillesMarverti, GaetanoBarocelli, ElisabettaMarycz, KrzysztofBarrajón-Catalán, EnriqueMarzocco, StefaniaBarriuso, JorgeMásson, MárBartelt, AlexanderMasullo, MariorosarioBasiak, EwelinaMateo, CesarBasini, GiuseppinaMati-Baouche, NarimaneBassin, PaulMatsugo, SeiichiBatista, IrineuMatsuura, HiroshiBavaro, Simona LuciaMattei, CesarBavestrello, GiorgioMaurer, StefanieBechmann, NicoleMáximo, PatríciaBeckel, J. M.May, AaronBedini, EmilianoMazur-Marzec, HannaBeemelmanns, ChristineMcCarthy, PumtiwittBeld, JorisMcculloch, MalcomBellich, BarbaraMcdougal, Owen M.Bełtowski, JerzyMcFeeters, RobertBerillis, PanagiotisMcPhail, KerryBerrue, FabriceMedvedev, Ilya NikolaevichBerry, JohnMehbub, Mohammad FerdousBertin, Matthew J.Mehta, PoojaBessa, LucindaMéndez, LucíaBezirtzoglou, EugénieMendonca, AntonioBiedermann, DavidMenna, MarialuisaBiernasiuk, AnnaMenzel, LorenzoBikiaris, DimitriosMeschwitz, SusanBishop, KarenMessyasz, BeataBisson, JonathanMetón, IsidoroBjørndal, BodilMeyer, Anne S.Blanton, CynthiaMeyerholz, David K.Blomme, EricMi, Fwu-LongBlumer-Schuettea, Sara E.Michalak, IzabelaBoddy, Christopher N.Micillo, RaffaellaBoehr, David D.Mickel, StuartBoekema, BoukeMiki, YasuhiroBogni, AlessiaMilledge, John J.Bohn, TorstenMiller Conrad, LauraBokias, GeorgiosMiller, Aaron W.Bondon, ArnaudMiller, John H.Bonferoni, Maria CristinaMilthorpe, BruceBongers, KaleMimaki, YoshihiroBorota, AnaMinato, Ken-ichiroBose, UtpalMinkiewicz, PiotrBosso, LucianoMishra, NigamBotana, Ana MaríaMitchell, Miguel O.Bottacini, FrancescaMiyaoka, HiroakiBouaïcha, NoureddineMiyashita, KazuoBoukouvalas, JohnMizuno, MasashiBourdelais, AndreaMłynarski, JacekBourguet-Kondracki, Marie-LiseMobbili, GiovannaBowden, BruceMocan, AndreiBoysen, ReinhardModica, Maria VittoriaBrandstetter, HansMolinaro, AntonioBrantsæter, Anne LiseMolinski, TadeuszBraquart-Varnier, ChristineMomper, LilyBrecker, LotharMong, Mei-ChinBreitenfeld Granadeiro, LuizaMoni, LisaBrestoff, Jonathan R.Moniuszko-Szajwaj, BarbaraBrinchmann, Monica FengsrudMontalvo-Rodríguez, RafaelBrkljaca, RobertMontero, Maria JoseBroberg, AndersMoon, MinhoBrück, ThomasMorais, Zilda BragaBrycki, BogumilMorales-González, José AntonioBuchet, ReneMoran, YehuBucolo, ClaudioMoreira, CristianaBulgariu, LauraMoreno, AndrésBumgardner, Joel D.Morganti, PierfrancescoBurcul, FrankoMori, KiyoshiBurger, MichaelMorikawa, ToshioBurghardt, KyleMorimura, ShigeruButtachon, SuradetMorita, NaokiCabral, JaydeeMorzycki, Jacek W.Cabrele, ChiaraMourelle, M. LourdesCaffrey, PatrickMukherjee, SomnathCafiero, MauricioMuller, ChristianCain, RickyMüller, Werner E.G.Calcabrini, CinziaMulloy, BarbaraCallery, Patrick S.Mummadisetti, ManjulaCammarata, MatteoMundt, SabineCampanale, Joseph P.Muramoto, KojiCampbell, ChristopherMurata, MichioCampbell, LeeMurphree, S. ShaunCampos, Samuel K.Murphy, Ciara M.Capon, RobMurphy, Patrick J.Caputo, Gregory A.Murugaiyan, JayaseelanCardullo, NunzioMuschiol, JanCarmeli, ShmuelMusso, IoanaCaro, YanisNagai, TakeshiCarradori, SimoneNagaraju, Ganji PurnachandraCarro, LorenaNah, Jae-WoonCarta, GianfrancaNaka, HiroakiCarvalho, PauloNakatani, YoshihikoCasapullo, AgostinoNandi, SaikatCascioferro, StellaNanjappa, DeepakCastellano, FlaviaNasopoulou, ConstantinaCastro, MariaNasri, RimCauli, OmarNatadra Tabudravu, JiojiCeranowicz, PiotrNaughton, LynnCerenius, LageNaughton, ViolettaCerra, BrunoNaumann, Todd A.Cestele, SandrineNencioni, LuciaĆetković, HelenaNett, MarkusChaimbault, PatrickNeumann, JakeChand, SubhashNeves, Adriana CunhaChang, Long-SenNewman, David J.Chankhamjon, PranatchareeyaNicoletti, RosarioChannappanavar, RudraNicosia, AldoChao, Chih-HuaNiedziela, TomaszChaubet, FrédéricNieto, FranciscoChecconi, PaolaNifantiev, Nikolay EChen, GuangyingNishimura, ShinichiChen, YiliangNofer, Jerzy-RochCheng, ZheNoguchi, TamaoChetwynd, AndrewNorte, ManuelChevrot, RomainNoshita, ToshiroChiang, Meng-TsanNtalli, Nikoletta G.Chiang, Tai-anNuzzo, GenoveffaChiesa, GiuliaObanda, DianaChobot, VladimirOberthür, DominikChoi, HyukjaeO’Doherty, GeorgeChoi, Jae SueOfford, JamesChoi, Jong-ilOh, Dong ChanChu, XiangpingOhno, OsamuChüeh, Anderly C.Ohshiro, TakashiCiardelli, GianlucaOhta, ShinjiCicciù, MarcoOishi, TohruCicero, ArrigoOjika, MakotoCigana, CristinaOjima, TakaoCirillo, GiuseppeOkesola, BabatundeCitores, LuciaOkino, TatsufumiClark, FraserOklu, RahmiClivot, HuguesOkunade, Adewole L.Cochis, AndreaOlejnik, AnnaColanzi, AntoninoOrchard, SandraCole, Kathryn E.Ospina-Millán, Claudia A.Coleman, Mitchell C.Ostrowska, KingaColey, Helen M.Otero, PazColl, JosepOtsuka, YuzuruComi, GiuseppeOves-Costales, DanielCongestri, RobertaOyama, FumitakaConlon, MichaelOzaki, TaroConway, JonathanPace, VittorioCopolovici, Dana MariaPagani, StefaniaCorsaro, Maria MichelaPalanisamy, Satheesh KumarCosco, DonatoPalenzuela López, José AntonioCosta de Morais, Rui ManuelPalmer, MichaelCosta, FernandoPalumbo, AnnaCostantino, ValeriaPang, BoCouzinet-mossion, AureliePantic, Jelena M.Crassous, JeannePapaccio, GianpaoloCremades Ugarte, JavierParikesit, Arli AdityaCueto, MercedesParisini, EmilioCzerwinska, MonikaParlesak, AlexandrD’Amora, UgoParolini, CinziaD’Anneo, AntonellaParrino, BarbaraD’auria, ValeriaPatrick, Kennerly S.Dal Ben, MatteoPattenden, GeraldDaly, NorellePavic, ValentinaDamalanka, VishnuPeana, Massimiliano FDamiani, ElisabettaPeifer, ChristianDante, SilviaPeigneur, SteveDaranas, AntonioPelin, MarcoDash, RanjeetPeltomaa, ElinaDavies, PeterPeng, TengDavis, Keith R.Pepi, MilvaDavis, RohanPerego, PaolaDawood, Mahmoud A.O.Pereira, DavidDe Agostini, ArianePereira, José AugustoDe Almeida Coelho De Sousa, Maria JoãoPereira, LeonelDe Caralt, SoniaPérez, María J.De Geest, BartPérez-Pascual, DavidDe Marco, RossellaPerin, GiorgioDe Mendonça, Dina I. M. D.Perinelli, Diego RomanoDe Morais, Rui Manuel Santos CostaPeriyasamy, PalsamyDe Pascale, DonatellaPersico, MarcoDe Rivero Vaccari, Juan PabloPescitelli, GennaroDe Vendittis, EmmanuelePestov, AlexandrDe Vries, RonaldPETER, Martin G.Delbarre-Ladrat, ChristinePetit, KarinaDella Sala, GerardoPetrova, Olga E.DellaGreca, MarinaPfeffer, Frederick M.Delmastro-Greenwood, MeghanPiccialli, VincenzoDennis, James E.Piemontese, LucaDeplazes, EvelynePiggott, AndrewDeslouches, BerthonyPiiparinen, JonnaDevy, JérômePikuła, MichałDhakal, DipeshPiluso, SusannaDhanji-Rapkova, MonikaPimienta, Rodney LacretD’hooghe, MatthiasPinto, Diana C. G. A.Diamond, GillPires, CarlaDiana, PatriziaPiret, JocelyneDibas, AdnanPlastina, PierluigiDíez, DavidPlaza, AlbertoDijkstra, Bauke W.Plażuk, DamianDilokpimol, AdipholPoater, AlbertDmitrenok, Pavel S.Poma, PaolaDolashka, PavlinaPomin, Vitor H.Domínguez, HerminiaPorter, AndrewDomínguez-Álvarez, EnriquePoterszman, ArnaudDonato, RosarioPozo, Óscar J.Dong, ShihuiPozzolini, MarinaDoriano Bianco, ArmandoPradhan, ShantanuDormond, OlivierPrentis, PeterDosoky, NouraProestos, CharalamposDouglas, TimothyProksch, PeterDuddupudi, AnanthaPruess, Birgit M.Duggan, Brendan M.Pucciarelli, SandraDuh, Pin-DerQuan, TaihaoDurán-Riveroll, LorenaQuesada Pérez, Víctor ManuelDurazzo, AlessandraQuiliano, MiguelDutertre, SebastianRaabe, GerhardDwivedi, ChandraharRadaram, BhaskerDyshlovoy, SergeyRadwan, MohamedDziubla, ThomasRaftery, RosanneEbrahim, WeaamRagg, EnzioEchevarria, JuanRahimi, FaridEder, KlausRahman, AzizurEhrlich, HermannRai, DilipEl Sayed, KhalidRajauria, GauravEldeeb, MohamedRaje, MithunEl-Demerdash, AmrRajpurohit, SurendraElleuche, SkanderRallis, MicaelEl-Seedi, Hesham R.Rämä, TeppoElshahawi, SherifRamachandiran, IyappanEnache, Mădălin-IancuRamaraj, PanduranganEscargueil, AlexandreRamsay, RonaEspada, JesúsRandazzo, AntonioEsteve-Romero, JosepRanzato, EliaEvans, Franklin D.Rash, Lachlan D.Ezquerra-Brauer, Josafat-MarinaRatovitski, EdwardFabiani, RobertoRaymond, JamesFalqué, ElenaRazani, BabakFang, Ronnie H.Rebuffat, SylvieFanizzi, Francesco PaoloRemesh, SoumyaFarrugia, BrookeRescifina, AntonioFelgueiras, HelenaReyes, FernandoFengying, TangRey-Stolle, FernandaFenical, WilliamRho, Jung-RaeFernandes, CarlaRigano, DanielaFernández, José JavierRiguera, RicardoFernando, BinoshaRijo, PatríciaFerraboschi, PatriziaRinge, DagmarFerreira, Paula M.Rita Costa-Pinto, AnaFerrieres, VincentRivero, SoniaFiedler, Hans-PeterRobb, CraigField, RobertRobinson, Samuel D.Figadere, BrunoRobles, LukeFilker, SabineRocha, Hugo A. O.Fisher, Jed F.Rocha-Martin, JavierFitton, HelenRodriguez, JaimeFlematti, Gavin R.Rogato, AlessandraFleury, YannickRoldo, MartaFloresta, GiuseppeRomano, GiovannaFocsan, Alexandrina L.Romano, NiclaFogacci, FedericaRomano-Chernac, Fabian B.Fontana, AngeloRomo, Maria Del Mar Ortega-villaizanFontés, MichelRorrer, GregoryForget, AurelienRose, Peter.Fouillaud, MireilleRossignol, JulienFranco, ChristopherRosso, Marie-NoëlleFranzke, Claus-WernerRousselle, PatriciaFreitas, AnaRóżalska, BarbaraFrosini, MariaRozhon, WilfriedFuchs, SabineRudrawar, SantoshFujii, RitsukoRufino-Palomares, Eva E.Fujii, TomomiRullo, RosarioFujimori, KoRupasinghe, ThusithaFujita, MasakiRussell, Fraser D.Gaascht, FrancoisRusso, PasqualeGabriel, KyleRusso, PatriziaGajjar, ChiragRussu, WadeGalanis, AlexisRyan, AliGalasiti Kankanamalage, AnushkaSabatier, Jean-MarcGalasso, ChristianSabel, TinaGaleazzi, RobertaSabila, PaulGales, LuísSacco, PasqualeGallieni, MaurizioSaez, CarmenGaluska, SebastianSaha, Sushanta KumarGambari, LauraSahariah, PriyankaGanesan, A.Saisho, YoshifumiGanjloo, A.Saito, YoshiroGarbayo, InésSakai, TakeoGarcía Linares, SaraSakamoto, ToshioGarcia Vaquero, MarcoSakilam, SatishGarcia-Estañ, Luis PerezSala, LucaGarcía-González, Carlos A.Sala, RobertoGard, PaulSalehi-Ashtiani, KouroshGardner, Paul D.Salud Bea, Roberto De LaGarikipati, DilipSalvador, AndreiaGaro, ElianeSanchez, GoarGaspar, DianaSánchez-Fidalgo, SusanaGasparri, FabioSanjust, EnricoGatti, LauraSankaran, Ganapathy SubramanianGavagnin, MargheritaSanmartin, ChiaraGeest, Bart DeSansone, ClementinaGeirsdottir, MargretSantini, AntonelloGendaszewska-Darmach, EdytaSantos, InêsGenta-Jouve, GrégorySapudom, JiranuwatGerdol, MarcoSarabia, FranciscoGerlach, JaredSashiwa, HitoshiGerothanassis, Ioannis P.Sasso, SeverinGiannini, CleliaSatake, MasayukiGibert, YannSatheesh, SathyanesonGill, HarsharnSato, EiichiGill, SantokhSato, MasaoGillevet, PatrickSato, ShigeruGilmour, JimSaurav, KumarGil-Ortega, MartaSaviola, AnthonyGiovine, MarcoSavion, NaphtaliGłąbska, DominikaSchiavone, MarcoGnavi, GiorgioSchlaepfer, Isabel RGoldman, Ellen R.Schmalz, Hans-GuentherGomes, Ana P.Schmid, JochenGomes, AndreiaSchmid, MatthiasGomes, NelsonSchmidt, MartinGomes, Nelson G.M.Scholz, BettinaGoncalves, OlivierSchröder, Heinz C.Gong, XiaomingSchroeckh, VolkerGonzález Maglio, Daniel H.Schroeder, ChristinaGorgoulis, VassilisSchubert, MarioGorrell, MarkSchuehly, WolfgangGotor Fernandez, VicenteSchulte, CarstenGotsbacher, MichaelSchulze, MargitGoudoulas, ThomasSchwartz, Stanley A.Gracia, IgnacioSeca, AnaGray, Christopher A.Seetharam, ArunGraziano, GuellaSeger, ChristophGreatrex, BenSerra, StefanoGrignani, GiovanniSerrano, AurelioGrkovic, TanjaSerrano, Dolores R.Grogan, GideonShaaban, Khaled AGruber, ChristianShakya, Viplendra Pratap SinghGudbrandsen, Oddrun A.Shang, ZhuoGuriec, NathalieShankar, EswarGurramkonda, ChandrasekharShaw, SubrataGushina, IrinaSheu, Jyh-HorngGutiérrez-Juárez, RogerShikov, Alexander N.Haarmann-Stemmann, ThomasShimada, YasuhitoHadjiargyrou, MichaelShin, JongheonHadwiger, LeeShinada, TetsuroHalama, AnnaShipman, KateHampel, DanielaShirahata, TatsuyaHan, Byung WooSholkamy, EmanHan, Jong WonShukla, ShrutiHano, ChristopheShuto, SatoshiHansen, EspenSiciliano, CarloHanski, LeenaSiewert, BiankaHanssen, Kine O.Sikazwe, DonaldHara, HiroshiSil, PayelHarder, JensSilipo, AlbaHarding, IanSilva, Pedro JorgeHarmanci, Arif OzgunSilva, TiagoHarrison, Paul H. M.Simpson, TJHartmann, AnjaSingh, ArunimaHarvey, Patricia J.Siodłak, DawidHashimoto, MasaruSita, GiuliaHassan, MohamedSleebs, Brad E.Hatae, NoriyukiSmedile, FrancescoHaug, Bengt ErikSmid, Scott D.Haug, TorŚmieszek, AgnieszkaHaverkamp, Richard G.Śmiga-Matuszowicz, MonikaHe, HongliangSmith, DavidHearn, Michael J.Smith, Thomas E.Helaly, SoleimanSmyrniotopoulos, VangelisHelbert, WilliamSolano, FranciscoHelesbeux, Jean-JacquesSolovchenko, AlexeyHenen, Morkos A.Sonntag, BettinaHerak Bosnar, MajaSorci, LeonardoHidari, KazuyaSotelo, CarmenHinderlich, StephanSoule, TanyaHindra, HindraSportelli, Maria ChiaraHirakawa, HidehikoSpurio, RobertoHlawaty, HannaSrivedavyasasri, RadhakrishnanHoffman, AngelaStacewicz-Sapuntzakis, MariaHofmann, JohannStará, AlžbětaHosono, MasahiroStefanidou, Maria E.Houen, GunnarStefanovic, BrankoHu, ChenlinStevens, ColeHuang, Chun-YungStockdill, Jennifer L.Humphries, BrockŠtrukil, VjekoslavHungerford, JamesSu, Jui-HsinHwang, Bang YeonSugahara, TakuyaHwang, Pai-AnSugawara, AkihiroHybertson, Brooks M.Sugiura, YoshimasaIanora, AdriannaSuh, Dae-yeonIbrahim, FandiSuleria, HafizIlan, MichaSumi, ShoichiroIlarraza, RamsesSung, Ping-JyunIlaš, JanezSung, Wang ChouImperatore, ConcettaSurguchov, AndreiInahashi, YukiSutherland, John B.Indiveri, CesareSvavarsson, Halldor GudfinnurInuki, ShinsukeSvetashev, VasilyInuzuka, ToshiyasuSvob Strac, DubravkaIoannou, EfstathiaŚwiątek, ŁukaszIshihara, KenjiSynytsya, AndriyIslam, M. NurulSztanke, MałgorzataIsnansetyo, AlimTaira, JunseiIto, TakuyaTakahashi, KenIvanov, Alexander VTakano, KatsuraIwasaki, ArihiroTalora, ClaudioIzawa, HironoriTanabe, YooJackson, Stephen A.Tanaka, JunichiJadhav, Gopal P.Tarman, KustiariyahJanczarek, MonicaTarr, Deirdre Ellen K.Jankauskaite, VirginijaTartaglione, LucianaJanowski, MiroslawTasdemir, DenizJansen, RolfTashima, ToshihikoJantas, DanutaTatulian, Suren A.Jaradat, NidalTava, AldoJennings, Jessica AmberTayebati, Seyed KhosrowJerebtsova, MarinaTebben, JanJerković, IgorTenorio, Manuel JimenezJesionowski, TeofilTeodoro, JoãoJimbo, MitsuruThe, ErlindaJiménez, CarlosThèvenod, FrankJoo, Hong-GuThomas, OlivierJug, MarioThomas, Y. K. ChanJun, Joon-YoungTimoshenko, Alexander V.Jung, Jee H.Tischler, DirkJusticia, JoséTommonaro, GiuseppinaJuszczak, LesławTomoda, HiroshiKaas, QuentinTopakas, EvangelosKadam, Shekhartori, MotooKadokawa, Jun-ichiTorikai, KoheiKafarski, PawelTørris, ChristineKalani, KomalTosti, ElisabettaKalinin, VladimirTotta, PierangelaKällén, BengtTribulova, NarcisaKamlage, BeateTrincone, AntonioKampranis, SotiriosTripathi, AshootoshKanakkanthara, ArunTrippier, Paul C.Kanekiyo, TakahisaTsafantakis, NikolaosKarentz, DenebTseng, Kuang-WenKarmakar, ParthaTsuchiya, YuichiKarpiński, Tomasz M.Tsuda, MasashiKaruso, PeterTsurkan, Mikhail V.Kashman, YoelTuccinardi, TizianoKasten-Jolly, JaneTudela, JoséKaszowska, MartaTulla-Puche, JuditKathuria, HimanshuTurk, TomKatikou, PanagiotaTurrini, EleonoraKato, Takamitsu A.Ulanova, DanaKavita, KumariUlber, RolandKawamura, AkiraUmerska, AnitaKawashima, HidekiUrban, PhilippeKayano, Shin-ichiUsami, YoshihideKeller, UllrichUsoltseva, Roza V.Kelley, ThomasUsui, TakeoKelly, MicheleUsuki, YoshinosukeKem, William R.Vale, Carlos Alberto Garcia DoKempaiah, PrakashVale, CarmenKerch, GarryVale, PauloKeyzers, Robert A.Valentao, PatriciaKhalil, Zeinabvan Berkel, WillemKhan, MohsinVan Der Merwe, MarieKhurshid, ChrowVan Lanen, StevenKikuchi, TakashiVázquez, José AntonioKim, Hee-SikVega Gutierrez, SarathKim, Hyun JoonVelu, Sadanandan EKim, HyungwooVenkatesan, JayachandranKim, Ki HyunVerestiuc, LilianaKim, LeungVerma, SurbhiKirienko, NatashaVetter, IrinaKirill, GolokhvastVetvicka, VaclavKłęk, StanisławVignes, MichelKlettner, AlexaVilanova, ManuelKlontzas, MichaelVílchez, CarlosKluza, JéromeVinogradov, EvgueniiKnölker, Hans-JoachimVlashi, ErinaKobayashi, YoshiroVllasaliu, DritonKolmar, HaraldVoliani, ValerioKomaki, HisayukiVon Knethen, AndreasKong, Chang-SukVon Schacky, ClemensKootala, SujitVorob’ev, Mikhail M.Kornprat, PeterWang, Bin-GuiKoshino, HiroyukiWang, PengchengKoskela, Harri T.Wang, QunKotoku, NaoyukiWang, San-LangKoutelidakis, Antonios E.Wang, XiaohongKovbasnjuk, OlgaWang, ZhihanKowalczewski, PrzemysławWang, ZhijunKozlowska, JustynaWase, NishikantKranz, MathiasWeber, David S.Krock, BerndWeber, J. MarkKuan, Yu-HsiangWeidenhofer, JudithKubala, LukášWeigmann, BennoKubatka, PeterWelsh, MichaelKubicova, LenkaWemheuer, FranziskaKubina, RobertWest, LyndonKubo, TaiWestcott, Stephen A.Kubota, TakaakiWhittington, CarlKudo, FumitakaWichard, ThomasKumar, ManojWie, Myung-BokKurihara, HideyukiWiedman, GregoryKurtböke, IpekWielgoss, SébastienKusano, ShuheiWilliams, DanielKwakowsky, AndreaWilson, DavidLa Clair, JamesWink, JoachimLa Regina, GiuseppeWińska, KatarzynaLaatsch, HartmutWioleta, KowalczykLabbozzetta, ManuelaWitulski, BernhardLabrou, NikolaosWłodarczyk, MaciejLacret, RodneyWlodawer, AlexanderLakshminarayanan, RajamaniWozniak-Knopp, GordanaLaner-Plamberger, SandraWright, AmyLang, SiegmundWright, ColinLaroche, CélineWu, BinLatorre, JessicaWu, Chang-JerLauder, BobWu, Chang-YiLaurent, FerriéWu, Chi-ReiLaurienzo, PaolaWu, Li-ChenLaustsen, Andreas HougaardXavier, Cristina Pinto RibeiroLavandera, José LuisYamada, ShuheiLavoie, SergeYamaki, KohjiLaws, Edward A.Yan, XiaohuiLazado, C. C. Yang, InhoLe, Hoang-ThanhYaoita, YasunoriLee, Bong HoYates, Edwin AlexanderLee, Hyeon YongYermak, IrinaLee, Jae KwonYotsu-Yamashita, MariLee, JaehwiYu, DiLee, Jong SukYu, Yan PingLee, Jong-SeokYuan-Bin, ChengLee, Jun SikYuasa, MasayoshiLee, KangwonYurchenko, AntonLee, Kuo-KauZabetakis, IoannisLee, Sang-HanZanolla, MarianelaLee, Yeon-JuZaucke, FrankLegros, ChristianZedler, Julie A. Z.Leitão, Jorge H.Zgórka, GrażynaLepiarczyk, EwaZhang, ChiLeporatti, StefanoZhang, RunLeshchenko, Elena V.Zhao, RuimingLestavel, SophieZhou, ShengbinLeung, IvanhoeZhou, ShuanhuLeverett, BetsyZhou, WenchaoLewinska, AnnaZielenkiewicz, UrszulaLi, Hsing-HuiZielińska-Pisklak, Monika A.Li, MiaomiaoZimba, PaulLi, WenyiZotchev, SergeyLi, XuwenZubía, EvaLi, Zijian


